# On the Distribution of the Blood-vessels in the Mucous Membrane of the Stomach

**Published:** 1851-11

**Authors:** Henry Frey


*On the Distribution of the Blood-vessels in the Mucous Membrane of the Stomach.* By Henry Frey.—The distribution of the blood-vessels in the gastric mucous membrane has an interesting relation to its double function; for the vessels of the surface, which are those most concerned in absorption, are veins, and have a large diameter; whilst those of the deeper portions of the membrane, which are subservient to secretion, are arteries, which form very delicate net-works around the gastric follicles.—*Ibid, from Henle and Pfeufer's Zeitschrift.*

